# Utilizing Artificial Intelligence to Facilitate Qualitative Surgical Research

**DOI:** 10.1097/AS9.0000000000000577

**Published:** 2025-05-21

**Authors:** Orly N. Farber, Corey M. Abramson, Amanda J. Reich

**Affiliations:** From the * Department of Surgery, Brigham and Women’s Hospital, Boston, MA; † Center for Surgery and Public Health at Brigham and Women’s Hospital, Boston, MA; ‡ Department of Sociology, Rice University, Houston, TX.

## INTRODUCTION

Artificial intelligence (AI) is increasingly discussed in the surgical literature for its quickly evolving clinical, educational, and research applications.^1,2^ Concurrently, qualitative surgical research has gained popularity, evident in analyses of publication trends^3^ and recently developed guides and commentaries focused on the application of qualitative methods.^4,5^ While there is some discussion of AI applications in qualitative research in health and social science,^6^ little crosstalk exists between the burgeoning conversations in AI and qualitative surgical research specifically. In this perspective piece, we argue that AI – the field that develops algorithms enabling machines to perform complex tasks, including simulating human reasoning – offers research applications that may be especially beneficial for qualitative surgical inquiries. We describe how thoughtful implementation of research strategies incorporating AI can facilitate rigorous qualitative explorations that yield valuable insights across the field of surgery.

## DEFINITIONS WITHIN ARTIFICIAL INTELLIGENCE

As AI applications surface in surgical research and practice, surgeons should have a basic understanding of the field’s terminology. Firstly, AI includes both discriminative and generative systems. Discriminative systems distinguish categories within data, while generative systems learn patterns and produce new data based on existing information. Machine learning is a subset of AI, which focuses on using existing data to identify patterns and improve task performance. Large language models (LLMs) are a specific application of generative AI, which use very large datasets to train computer systems to understand and produce human-like text, enabling them to perform a variety of language tasks (eg, discrimination, generation, translation, summarization, coding, and question-answering). Lastly, generative pretrained transformers are a subtype of LLM developed by OpenAI.^1,7^

## THE CASE FOR ARTIFICIAL INTELLIGENCE IN QUALITATIVE SURGICAL RESEARCH

Qualitative methods allow for different modes of inquiry than quantitative methods but are less commonly found in surgical publications. Qualitative approaches focus not on measuring outcomes, but rather, exploring and explaining phenomena through careful observation. While qualitative methods have gained ground in the surgical literature in recent years, they remain underutilized in part because of concerns with their rigor and reproducibility.^3,8^ An analysis of publication trends of qualitative surgical studies performed before 2015 found that nursing journals published nearly half of the studies identified while surgical journals only housed around 8% of the papers.^3^

While formal explanations to account for the paucity of qualitative research in surgical journals remain limited, potential reasons include: (1) The time-consuming nature of collecting and analyzing qualitative data coupled with surgeons’ heightened time constraints, (2) Diminished incentive to conduct qualitative research given the lower likelihood of successful publication in a surgical journal, and (3) Less exposure to and familiarity with qualitative methods within the field of surgery.^8,9^ AI applications, as we will describe, have the potential to ameliorate some of these concerns namely by accelerating qualitative data collection and analysis and providing support to researchers pursuing qualitative approaches.

## ARTIFICIAL INTELLIGENCE USE IN QUALITATIVE RESEARCH

AI applications afford a range of opportunities to facilitate qualitative analysis. In Table 1 we outline some of these opportunities – many of which are in their early stages of application – for AI implementation. Notably, AI can be used to facilitate research design, data collection and cleaning, checking for errors, analysis, and the writing and presentation of qualitative findings. Some of the software platforms often used for qualitative analysis, such as ATLAS.ti and NVivo, now offer built-in AI tools to automate coding. This can facilitate both efficient indexing and mining of text to identify patterns. Additionally, social scientists have used AI to scale up qualitative studies to include more data, de-indentify subjects, and identify patterns. While qualitative projects often rely on small samples due to the intensive nature of data collection and analysis, AI can expand projects by aggregating data from wider pools of participants, rapidly de-identifying data, and helping analyze large numbers of field observations or in-depth interviews.

**TABLE 1.** Existing Applications and Theoretical Opportunities for AI in Qualitative Research

Phase of Research		Examples
Research design	Literature review	• Utilizing LLMs to identify key studies and to facilitate systematic review
	Interview guide development	• Inputting drafts of interview guides into LLMs to help revise and simplify language before pilot testing
Data collection	Conducting interviews	• Employing LLMs to conduct structured written interviews with participants
	Tracking experiences	• Employing LLMs to remind patients to track their experiences (eg, symptoms), provide platforms for interactive journaling, and record the response
Data cleaning	Data processing	• Using AI-assisted transcription services to transcribe audio or check for transcription accuracy or • Using AI to reconstruct damaged audio or video
	De-indentification	• AI-facilitated de-identification to remove personal identifiers from transcripts
Analysis	Memos and summaries	• Utilizing LLMs to help create summaries of documents, transcripts, or other text
	Codebook generation	• After inputting interviews or fieldnote transcripts into an LLM, AI can help identify potential codes for human consideration
	Coding	• Analytic software such as ATLAS.ti and NVivo offer AI tools to automate coding
	Theme identification	• AI tools can facilitate the identification of themes from coded text
Presentation	Data visualization	• LLMs can help generate tables, graphs, or offer suggestions for data visualization
	Writing	• AI can facilitate writing by creating outlines, editing inputted drafts, and checking for factual errors and inconsistencies

The following are suggested best practices for and potential pitfalls of incorporating AI into qualitative research. We conducted a thorough literature review and found a variety of applications but few guidelines, prompting us to develop these recommendations. Our suggested practices are the collective perspective of the authors who have varied and combined experiences in surgery, qualitative research, and AI.

Best practices: The 5 Ts for applying AI in qualitative studies

- Tandem use: Use AI modalities as an adjunct, not as a replacement for researcher-driven design, data collection and cleaning, analysis, or presentation.
- Targeted application: Use AI modalities for a defined and confined purpose. Consider when traditional analytic methods may be preferred.
- Training/testing: Ideally, train and fine-tune models on relevant data. Do not only rely on public-access LLMs (eg, ChatGPT), but consider adopting application-specific LLMs that recognize language in the appropriate context. However, acknowledging that this may not be feasible for most investigators, test by evaluating the AI tools and identifying discrepancies between human and AI approaches/outputs.
- Triple check: Exercise diligence in evaluating findings. If the outputs from LLMs are unusual or inconsistent, crossreference the AI-generated output by reading original transcripts, generating summaries, or coding manually. Additionally, ensure AI-facilitated procedures are replicable.
- Transparency: Include descriptions of AI utilization in methods and limitations. Explain in detail the ways AI was employed within the study, and what limitations this application might impose.

Potential pitfalls in applying AI to Qualitative research

- Hallucination: LLMs can create outputs that are not real. For qualitative studies, this can include composits (eg, combining quotes) or fabrications (eg, generating plausible but false statements). This pitfall can be mitigated by fine-tuning or domain-specific training of models, thorough fact-checking, iteration, and crossreferencing outputs with source data.
- Bias amplification: Algorithms can inherit biases from their training data and reflect analyst decisions. In healthcare, AI tools can worsen disparities if not used with care. This pitfall can be mitigated by targeted applications and crossreferencing.^10^
- Authorship concerns: Authorship concerns can arise when using AI to facilitate writing papers for dissemination. This can be avoided by transparency and tandem use. LLMs should not write entire papers, but rather AI tools can be used to help outline, revise, check prewritten text, and help identify missing references.
- Data security: Issues of data security can arise when using LLMs with human subjects’ data. Care must be taken to store data on secure servers and to avoid inputting patient information into nonsecure systems.
- Danger in scientisim: While AI can help facilitate and automate some aspects of qualitative analysis, it should not be treated as a more “scientific” approach to this work. High-quality qualitative research necessitates human interpretation and judgment.

## CONCLUSION

AI tools can supplement and enhance qualitative surgical inquiries. As the fields of AI and qualitative surgical research continue to grow in parallel, integration of AI-based methods will become inevitable. Thoughtful application of AI is necessary to ensure rigorous and ethical research conduct and has the potential to help uncover important insights in surgical education, research, and practice.

## ACKNOWLEDGMENTS

We thank Dr. Elizabeth J. Lilley for identifying the need to develop guidelines for AI applications in qualitative surgical research and for sharing her experiences and expertise.
